# The Nursing Supplement

**Published:** 1890-03-29

**Authors:** 


Being the Extra Nursing Supplement of "The Hospital" Newspaper.

All Contributions for this Supplement should be addressed to the Editor, The Hospital, 140, Strand, London, W.C., and should have the word "Nursing" plainly written in left-hand top corner of the envelope.

### En Passant.

**Bristol District Nurses.**—On the 18th the annual meeting of the Bristol District Nurses' Society was held, the High Sheriff (Mr. Lockley) in the chair. It looks as though the society overworked its nurses, or else does not choose strong, suitable women, for some of them have been ill all the year, and at the time of writing the report three were away for their health. Two new districts have just been opened, St. Matthias (Nurse Cecilia Ocker) and St. Michael (Nurse Annie Rendle). The Lady Superintendent was warmly thanked for her efficient services, and Dr. Parker for kindly lecturing to the nurses. The Treasurer's balance-sheet for the year showed an income of £974, and an expenditure of £1,049, leaving a deficit of £75.

**The Brassey Holiday Home.**—The above Home will be opened on the 3rd of April for the reception of nurses and their lady friends. The terms will be 15s., and we are glad to see this is made inclusive, and that cheap railway fares will probably be granted those going to the Home. We sincerely wish the Home every success, and hope an effort will be made to make it as little of an institution as possible. The rule "punctuality to prayers and meals absolutely necessary," is bad both in spirit and expression. What are these prayers that nurses of all shades of opinion are bound to attend? Or is the Home a sectarian establishment? We shall be glad to hear from Sister Frost on these points, as we are sure she is doing her best for the Home, and our criticism is offered in a friendly spirit.

**Short Items.**—There is some just indignation amongst the dissenters of Bath, because one of the qualifications for the post of matron to the United Hospital is that she be a member of the Church of England.—Mr. Burdett addressed the nurses of Marylebone Infirmary last Monday evening.—We are requested to state that the Nurses' Club at 12, Buckingham Street, Strand, numbers over 200 members, and that statements to the contrary are untrue.—A dramatic performance in aid of the Nurses' Club will be given on April 28th. Readers please hold themselves disengaged for that date, and we will give full particulars next week.—Sister Rose Gertrude has arrived at Molokai.—Miss Mollett and three nurses have arrived and are working hard at Johannesberg.—The Queen Victoria Jubilee Institute at Edinburgh have obtained a new Home for their nurses at 29 and 30, Castle Street.—The nursing staff of Ingham Infirmary are all invalided, it is believed from an escape of sewer gas.

**Thousands of Pounds for the National Pension Fund.**—We have the pleasure to acknowledge the following sums towards the £14,000 now being raised to at once bring the Donation Bonus Fund up to £40,000. We hope to be able to acknowledge contributions amounting to £1,000 every week until the £40,000 are completed. Contributions of any amount may be sent to the Editor of The Hospital, or to the Manager, National Pension Fund for Nurses, 8, King Street, Cheapside, London, E.C.:

Tenth Thousand.

| | £ | s. | d. | | £ | s. | d. |
|---|---|---|---|---|---|---|---|
| Goldsmiths' Company... | £500 | 0 | 0 | Lord Grimthorpe ......... | £50 | 0 | 0 |
| Messrs. Stern Bros. ...... | 150 | 0 | 0 | Mr. F. D. Mocatta ...... | 25 | 0 | 0 |
| Mr. W. S. Deacon......... | 100 | 0 | 0 | Messrs. Medwin & Lowy | 10 | 10 | 0 |
| Messrs. W. Greenwell & Co. ...................... | 1C0 | 0 | 0 | Mr. W. R. Rickett ...... | 10 | 0 | 0 |
| Messrs. Bircham & Co. | 52 | 10 | 0 | Miss Janet C. Topping | 2 | 2 | 0 |

Amount already received **Ten Thousand Pounds.**

**Frome Nurses' Home.**—The balance sheet of this institution shows an income of £423, and a balance carried forward of £11. Both private and district nurses are supplied, and the Mothers' Aid branch sends a certificated midwife to attend poor married women. There is also a Sick Comfort fund, and a reserve fund for providing increased house accommodation. Altogether the wants of the sick of Frome seem well attended to by this institution.

**The First Thousand.**—The question of smart uniforms and spare time in June is occupying the minds of the First Thousand, for the ever-gracious Princess of Wales has consented to distribute the certificates to the First Thousand Nurses who joined the Pension Fund, at Marlborough House some day towards the end of June, and it would indeed be a pity if any of those entitled to be present were unable to attend. The gathering ought to be a very pretty and a very representative one, and the uniforms will be a special point of interest.

**A Good Idea.**—The annual report of the Mitchell Home, Torquay, comes to us with a prominent green leaflet attached to the cover, and this is what the green leaflet says: "Pension Fund.—The Committee of the Torquay Nurse Institution think it would be most desirable to join the National Pension Fund, and they will be grateful for help to enable them to assist in this satisfactory manner those nurses, who, after many years of faithful service in the institution, leave through increasing years or sickness. The Committee think this would be a fitting and just testimony to the good work done by the nurses, especially by those working in the districts where their work is necessarily often most trying from exposure to all kinds of weather, and the older nurses require more consideration than the younger ones, as the premium for a nurse advanced in years is too high for her to pay unaided." Unless we are much mistaken the nurses of Torquay will get their pensions, and we advise other institutions to attach a similar green leaflet to their annual reports.

**American Notes.**—A delightful bundle of *Nightingales* has reached our office, from which we glean some notion of how our nursing sisters across the Atlantic are working. One quaint item of news is that six graduates of the Bellevue Training School for Female Nurses are now acting as instructors at the Mills School for Male Nurses. The Boston Training School issues its tenth annual report, and states that twenty-five nurses graduated last year on the completion of their two years' course. Miss Brown is Lady Superintendent.—The Brooklyn Nurses' Directory is under the Kings Co. physicians, and two or three ladies are on the board. The fee is 5 dols. to become a member, or rather to register, and 2 dols. each succeeding year.—An account of the Catholic Sisters who for many long years have quietly and efficiently administered the Leper Hospital of Tracadie was given lately in the *Montreal Star.*—The St. Luke's Hospital at Chicago has adopted an outdoor uniform or grey circular cloak, grey bonnet and veil. This is a significant move on the part of independent American women.—The American Nurses' Association, besides initiation fees, demands 5 dols. annually from its members. This shows that nurses are better paid there than here, for an English nurse could not afford to pay such a sum. The *Nightingale* sends a friendly greeting to The Hospital, which we return most heartily.

### Notes from Australia.

(*By Our Own Correspondent.*)

MELBOURNE, January 18th.

OUR new Governor had not been twenty-four hours in the colony, before he was assailed with a shower of begging letters, which luckily His Excellency had the sense to send on to the Charity Organisation Society. So far Lord Hopetoun has turned his attention to educational institutions chiefly, but we hope to interest him in our great hospital problem soon.

THE Hospital Sunday Fund for the current year has been dealt with, cheques being issued to the medical charities in the following order: Melbourne Hospital, £2,985 4s. 2d.; Alfred Hospital, £1,992 7s. 6d.; Benevolent Asylum, £872 2s. 10d.; Women's Hospital, £954 0s. 10d.; Hospital for Sick Children, £1,396 11s. 6d.; Eye and Ear Hospital, £548 17s. 3d.; Homœopathic Hospital, £883 3s. 2d.; Austin Hospital, £533 1s. 1d.; Immigrants' Aid Society, £384 11s. 8d. The institutions which received special votes were the Convalescent Home for Women, £100; Convalescent Home for Men, £100; Collingwood Dispensary, £100; Richmond Dispensary, £50. The total sum voted was £10,900.

A VERY sad case of a family almost exterminated by diphtheria has just occurred. Mr. William Heron Steel, engineer, contracted diphtheria. Three of his children caught the dread disease from their father, and rapidly succumbed, two of them dying on the 21st ult., and the third on the 23rd. Mr. Steel in the meantime was progressing as favourably as his medical attendant could hope. He appeared to have passed the most dangerous period of the attack, when, in spite of all precautions that were taken to keep the unwelcome news from him, he surmised that in the case of his children the contagion had proved fatal. On Christmas morning he attempted to sit up in bed, but the slight motion affected his heart, which was weakened by the disease, and he fell back unconscious. Stimulants were immediately applied, and he recovered slightly, but never gained sufficient consciousness to recognize any of his friends who had gathered round his bedside, and, rapidly falling again into a state of coma, he quietly breathed his last.

THE town of Broken Hill, which has been called into existence by its great silver mines, will soon become the wonderland of western New South Wales. It is a big town. In a year or two it will be a city. The *Argus* says its hospital is an institution of which Broken Hill has a right to be proud. It is a handsome stone building, situated on a low hill in the western suburb. It has beds for 40 patients, and is excellently provided for in other necessary ways. The institution is supported by subscriptions and contributions, supplemented pound for pound by the Government. As may be imagined the funds fluctuate considerably. Some good examples of liberality may be noticed. The Broken Hill Proprietary Company, on being appealed to, gave £500, and the mine directors, then on a visit to the Hill, added £90 to that from their own pockets. Following this Mr. Geo. M'Culloch, one of the original syndicate of the big mine, finding that it would cost £700 to furnish the hospital, sent in a cheque for that amount, and a ward now bears his name in commemoration of the gift.

THERE is very little purely nursing news this month; things are still unsatisfactory at the Melbourne Hospital, and satisfactory at the Alfred Hospital. At the last-named the half-yearly examination of the pupils of the Nurse Training School was held this week by Dr. W. H. Embling, Dr. M. Barclay Thomson, and Dr. R. E. Schlesinger. Ten nurses passed the final examination (two with credit), and nine the preliminary examination (one with much credit).

CONVERSATION is all about the Congress of the Australian Association for the Advancement of Science, at which some interesting medical papers have been read. Mr. Alfred J. Taylor, Hobart, read a paper on "Some Facts and Figures Relating to Vaccination." He said the protection against small-pox afforded by vaccination has been so fully demonstrated that it seemed almost like waste of time to add further proof to that already given of its efficiency. He quoted figures and facts showing the protective influence of vaccination, and said the only danger from vaccination was one for which its opponents were directly responsible. They shook public confidence in the operation, and as a consequence it was neglected until there was a scare, and then arose the danger that it might be performed hurriedly, carelessly, and without due regard to conditions. Dr. G. A. Syme read a joint paper, by Messrs. J. M. Smail and W. L. de L. Roberts, C.E.'s (Sydney), on "Purification of Sewage." Mr. Smail, in his part, said the various systems which had been tried for purifying sewage might be classed as filtration, chemical treatment, and irrigation. In all the towns that had, in the past, attempted to purify the sewage by chemical treatment, the greater number had abandoned the process for one of disposal and filtration over land. Other interesting papers were read on "Town Sanitation," "School Hygiene," and "Health Legislation," but in these points England has nothing to learn from Australia.

At a meeting of the committee of management of the Melbourne Hospital, the medical superintendent stated that the hospital was very much crowded. Some 66 cases, 40 of them fever and about half of that number typhoid, were refused admission during the past fortnight. There were at present 67 cases of typhoid fever in the hospital. The newly-appointed matron reported that, while the older nurses in the hospital were doing splendid work under discouraging circumstances, more accommodation for nurses was urgently required. It was suggested that the night staff of nurses might be accommodated in some building outside, but close to the hospital. Many of the nurses were doing housemaids' work, and this could not be remedied until the accommodation was increased so as to make room for some 20 pupil nurses. The report was referred to the Building Committee.

### Nursing Lectures.

By PERCY LEWIS, M.D.

### LECTURE VI.—THE ABDOMEN.

The abdomen is the largest compartment of the body, and contains most of the digestive organs. It is bounded behind by the spine and back muscles, in front by the abdominal muscles, at the sides by the ribs and abdominal muscles, above by the diaphragm, and below by the pelvis and its contained organs. The abdomen is in popular language called also the stomach. This is a mistake, for the stomach is only one of the organs contained in the abdomen, and its size is comparatively insignificant. It is placed in the upper part of the abdomen and only coming in contact with the abdominal walls at a part called, in popular language, the pit of the stomach, i.e., between the angles of the ribs, and at the left side, especially when distended, it is in contact with the ribs. If you put your left hand on your ribs about a hand's breadth above the hip bone you will just about cover this latter part. In medical language the pit of the stomach is called the "epigastrium."

The stomach is a hollow organ composed chiefly of an outside muscular coat and an inside lining called "mucous membrane," containing the small glands which make the gastric juice. At one end is the opening of the œsophagus or gullet by which food comes from the mouth; at the other is the opening of the small intestine. When empty the stomach has a capacity of about a pint, but it may be distended by food to hold three or four more. The œsophagus, stomach and intestines are spoken of collectively as the "intestinal canal"; they measure about thirty feet. The object of the length is to increase the surface of absorption, a surface still further augmented in the small intestine by minute velvety processes called "villi" with which the whole mucous membrane is covered. The small intestine, which is the longest part, is divided into three—the duodenum, the jejunum and the ileum. Like the stomach and large intestine it is composed of muscular coats and mucous lining, with multitudes of little glands.

Into the duodenum open the ducts of the liver and pancreas. The ileum or third part is the seat of ulcers in typhoid fever of which you will hear more later on. It ends in the large intestine in a valve called the "ileococcal," so arranged that food can go from the small into the large, but not vice versa. The small intestine occupies chiefly the middle part of the abdomen. The large intestine commences close to the right hip bone in a rounded end called the cœcum, into which the ileum opens; from this it goes straight up to the ribs—ascending colon—crosses the abdomen above the umbilicus—transverse colon—and then descends to the left hip bone—descending colon. There it makes a twist called the sigmoid flexure, and there passes into the pelvis when it is known as the rectum. In order that the intestines may move on each other without friction, they and the various other organs in the abdomen are covered, like the lungs and heart, with a smooth shining membrane, in this case called the peritoneum.

The liver is the large gland which manufactures the bile. Situated under the diaphragm, chiefly on the right side, it has attached to its under surface the gall bladder, where the bile secreted is retained until wanted. For nursing purposes the upper limit of the liver is found by drawing a line from the lower extremety of the sternum transversely to the right; the lower limit being about a hand's breadth below this. You may be told to apply leeches or poultices to the liver, so should know where to put them. The pancreas is so far away from the surface, that for nursing purposes it may be neglected.

I have now given you a very rough sketch of the anatomy of the organs of digestion and will now pass on to the physiology of them. The object of food is to nourish the different organs, that is, to repair the waste which is continually going on from use. The process by which food is turned into a condition in which it can be taken into the blood is termed digestion. The organs we are discussing are the digestive organs. Food is divided into the following classes: starches and sugars, as bread, vegetables, sugar; albumins, as meat, fish, curds of milk; fats, as butter, fat, cream; salts, as common salt: extractives, as beef tea, coffee, tea, and water. Now it is necessary for health that each of these classes should be represented in one's diet and must be digested in order to be of any use. The different classes get affected in different ways in different parts of the intestinal canal. In the mouth all the solids get more or less broken up by the teeth and softened with the saliva. But the saliva has another action; it changes the starch, which cannot be absorbed, into sugar which can; in fact digests it. In the stomach the fats get changed into oil, and the meat gets partially dissolved by the action of the gastric juice, or, rather, by its active principle, called pepsin. After a longer or shorter stay in the stomach, according to circumstances, the food is passed on, first the liquid and then the solid, into the small intestine. Here it meets the bile which acts chiefly on the fats and dissolves them; also the pancreatic juice which acts on the starches like saliva, and on the albumins like gastric juice; thus by the time the food reaches the large intestine it is nearly all liquid. Although there has been absorption of the water taken with the meal all the way along the stomach and small intestine, yet the amounts of the gastric and pancreatic juices and of the bile are so large that the food or "chyme" is always liquid at the ileocœcal valve. The chyme does not even represent all the solids of a meal, for they also have been partly absorbed on the way down. In the large intestine the remainder of the liquid is absorbed and the indigestible and undigested matter goes on to form the fæces or motions.

The intestine has also another function, it excretes; that is, separates from the blood substances which are no further use to it. This fact is taken advantage of in kidney disease and in the treatment of bilousness and headache, where the excreting function of the intestine is increased by purgative medicines.

### Everybody's Opinion.

[*Correspondence on all subjects is invited, but we cannot in any way be responsible for the opinions expressed by our correspondents. No communications can be entertained if the name and address of the correspondent is not given, or unless one side of the paper only be written on.*]

### MEUM AND TUUM.

EPHRAIM writes: Allow me to give expression to the great pleasure and satisfaction with which I read "Handclasp's" reply to "Justine's" letter, both of which have appeared in the Nursing Supplement. Reflecting, as "Justine's" letter did, upon the management of wards, the treatment of patients, and the character of the nurses in charge of them, it would have been unfortunate if it had been allowed to pass without observation, and especially was it desirable that it should be answered by some other nurse, and that it should not be allowed to go forth unchallenged as representing what nursing is, and what nurses are. If nurses are to be held in respect, and if the status of nurses is to be raised, such letters as "Justine's" will not promote these ends. As one who is interested in the question, and who has many opportunities of observing nurses, I am glad to be able to say that whilst many persons not possessed of the necessary mental and moral qualifications take up with nursing, and have no higher motive than "merely to gain a respectable living," I can bear willing testimony to the higher motives by which many of them are animated. Sympathy with the sick and suffering, womanly tenderness and purity of motive, a sense of responsibility, and unselfish devotion—these are qualities which should distinguish every nurse. Let me beg all nurses to read "Handclasp's" letter again, and ponder over it.

---

NURSE COLE writes: "Handclasp" treads on the toes of so many of her fellow-nurses in her letter to-day, that she must not wonder if some of us cry out for consolation to our constant friend THE HOSPITAL. Some of us do enter a hospital purely from motives of "love and devotion to the sick." No doubt to such the work comes easily, and they are generally clever nurses, beloved of their patients. Some of us enter a hospital, as some doctors do, because we are obliged to work, "merely to gain a respectable living." Of such "Handclasp" expects "instances of gross neglect and wicked indifference" not uncommonly! Love and devotion to any object cannot be voluntary—I mean. of course, as emotions constituting a motive. Fidelity to duty, even to a duty undertaken "merely to gain a respectable living," is possible to all honest women, and may develop as much "love and devotion for the sick" as the natural emotion occasions in different natures. "Tis said, best men are moulded of their faults," and the constant suppression of self involved in the faithful discharge of duties uncongenial to natural taste, ought to foster that spirit of unselfishness so necessary in a nurse; more especially as no trace of the repugnances felt can be suffered to appear in look or tone. Nowadays, again, some women turn from a fruitless search after absolute truth

"in earth or sun,
In eagle's wing or insect's eye,
Or in the questions we may try,
The petty cobwebs men have spun;"

to seek it in that life where "duty" is plain, and provides honest occupation for hand, heart, and brain; where, at least, they can practically love the "brother whom they have seen." Reasoning that if there is a "voice" speaking from God to man, those who try to live daily all the truth they know will surely hear it. To such the details of hospital life are often singularly repulsive, they having generally lived more in the subjective than the objective, and being unused to contact with pain and loathsome forms of disease. Yet can natural emotion, or the necessity of gaining a living, be so constant and powerful a motive for honest, loving care of the sick as the daily endeavour to "walk in the truth,"

as the final effort to find light? Let no woman turn aside from following up such an inspiration because the emotions of "love and devotion to the sick" are wanting! It would be interesting if our Editor would invite every nurse who takes THE HOSPITAL to state in one brief sentence "why she became a nurse." If he followed that up by investigating a few cases taken haphazard, "Handclasp" might be convinced that her opinion has been too hastily formed.

### Death in our Ranks.

NURSE FURMEDGE.—We recora with regret the death of Miss Maud Furmedge, aged twenty-eight, on March 6th. Miss Furmedge was a native of Gloucester, and had been connected with the Falsgrave Trained Nurses' Home, and had lately acted as a professional nurse at Scarborough. Latterly she was occupied with a serious case, and in her few spare moments gave her attention to a patient suffering severely from the influenza epidemic. Incessant work, together with loss of proper rest, could have but one result, and early in the week Dr. Taylor noticed the worn-out condition of Miss Furmedge. He saw at once that she was indisposed, and thinking she needed prompt and careful attention, he very kindly had her removed to his own residence in Queen Street. Here all that medical skill and efficient nursing could effect was done, but an attack of acute pneumonia supervened, to which on Thursday night, the 6th inst., Miss Furmedge succumbed. Wreaths were sent from Sister Dora, Dr. Arthur Butt, Dr. Godfrey, Dr. Everley Taylor, Nurse Clarson, and many other sympathisers.

NURSE EMILY RUNDLE.—It is with the most sincere regret we chronicle the death of Nurse Emily Rundle, aged 27, who died at the Firs Home, Bournemouth, on February 12th, from consumption. Nurse Rundle had worked at the Liverpool, Stoke-on-Trent, and Jersey institutions, and was greatly beloved by her fellow-nurses. Only last autumn, while lying ill at Bournemouth, she dressed us two sets of dolls, representing the nurses of the Jersey Institution, and of the Firs Home, Bournemouth, where Nurse Rundle was faithfully tended during her decline.

### Examination Questions.

FOUR very good answers were received this month, one from Oxford, one from Richmond, one from Dublin, and the best from Nurse Eva Brackenbury, of Downham Market, Norfolk. To her we have sent Dr. Fernie's book on "Influenza," and her answer will be printed next week. The question for April is: "Name five of the most common disinfectants in general use, and mention the purposes for which each is most useful." Answers, which must be short, and written on one side of the paper only, must reach our office by April 12th. We hope there will be more competitors this month, not for the sake of the prize, but because the mere effort of writing down such points must fix them on a nurse's memory. We are disappointed that more infirmary and district nurses do not compete; most of the answers come from hospitals.

### Appointments.

[It is requested that successful candidates will send a copy of their applications and testimonials, with date of election, to THE EDITOR, The Lodge, Porchester Square, W.]

GRIMSBY HOSPITAL.—We understand that Sister Shepherd and Miss Hoyle have been appointed respectively matron and nurse at Grimsby, where they will have to carry out the reforms to be inaugurated on March 31st.

### Notes and Queries.

TO CORRESPONDENTS.—1. Questions may be written on post-cards. 2 Advertisements in disguise are inadmissible. 3. In answering a query please quote the number. 4. If a private answer is desired, a stamped addressed envelope must be forwarded.

NOTICE.—We must really ask our correspondents to be more particular in enclosing name and address. Constantly we receive queries or letters with no name attached.

### Answers.

*N. E. F.* must send name and address.

*Nurse Mildred.*—St. Paul's Home, 62, Via Palestro, Rome.

*Glenn.*—The After-Care Association for Friendless Female Convalescents on Leaving Asylums for the Insane. The Drive, Walthamstow, Essex.

### Vacancies.

THE following vacancies are announced:

### Matrons.

Fulham Union; £80.

Kidderminster Infirmary.

Ophthalmic School, Hanwell; (deputy.)

Royal Albert Edward Infirmary, Wigan; £60.

Rochdale Infirmary; £40.

Stockton-on-Tees Hospital; £60.

### Nurses.

Abbey Poorhouse; £22
Aimee Nursing Homes.
Bournemouth Institution; £25.
Burton-on-Trent Institution; £25.
Blackheath Institution.
Bedford Infirmary; £20.
Bedfordshire Institute; £23.
Borough Hospital, Birkenhead.
Cancer Hospital, S.W.; £25.
Cottage Hospital, Harrow-on-the-Hill; £30.
Cottage Hospital, St. Helen's; £20.
Christ's Hospital, Hertford.
Coventry Hospital (night); £20.
Cardiff Infirmary; £24.
Central London Sick Asylum; £20.
Chester Deaconess House; £24.
Eastbourne Sanatorium; £30.
Greenwich Union, £27.
Greenock Poorhouse; £25.
General Nursing Institute.
Gainsboro' Nursing Association.
Hanover Institute, W.; £25.
Huddersfield Infirmary; £24.
Her Majesty's Hospital; £25.
Hull Nurses' Home.
Jessop Hospital; £20.
Kent and Canterbury Hospital (night); £22
Kent Institution; £30.
Liverpool Eye Infirmary; £20.
Lowestoft Hospital (night); £20.
Mrs. Elcock's Home; £25.
Mauldeth Hospital for Incurables; £30.
Mr. Wilson's Institution.
Massachusetts; £40.
North-Western Fever Hospital; £30.
North-West London Hospital; £24.
Ophthalmic School, Hanwell; £18.
Pendlebury Children's Hospital; £20.
Rivers Street, Bath.
Swansea Institute; £25.
St. Saviour's Infirmary; £20.
Shoreditch Infirmary; £20.
St. Vandas Nursing Home, Bromley.
Stamford, Rutland and General Infirmary; £25.
Stockport Infirmary.
Tewkesbury Hospital (night).
Tunbridge Wells Hospital; £25.
Victoria Park Hospital; £20.
Victoria Hospital, Glasgow (night).
Westminster Ophthalmic Hospital; £20.
West Kirby Nurses' Home.
Whitechapel Infirmary; £20.

### District Nurses.

Burton-on-Trent Institution.

Birmingham.

Reading.

Queen Victoria Institute, Edinburgh.

Upper Norwood.

### Probationers.

Burton-on-Trent Infirmary.
Burton-on-Trent Institution.
Children's Sanatorium, Southport.
Leeds Nurses' Institution.
Lancashire Infirmary, Peterborough
Lowestoft Hospital.
Notts Nursing Association.
Wirral Children's Hospital.

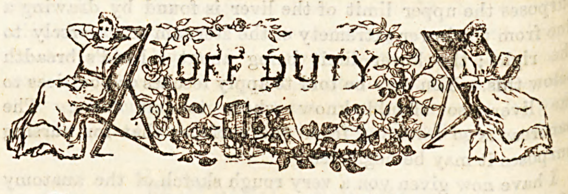

### Amusements and Relaxation.

N.B.—New Word Competition Commences April 5th, 1890, ends June 28th, 1890.

**FOURTH QUARTERLY WORD COMPETITION**

Commenced Jan. 4th, 1890, ends March 29th, 1890.

Three prizes of 15s., 10s., 5s., will be given for the largest number of words derived from the words set for dissection.

Proper names, abbreviations, foreign words, words of less than four letters, and repetitions are barred; plurals, and past and present participles of verbs, are allowed. Nuttall's *Standard* dictionary only to be used.

N.B.—Word dissections must be sent in WEEKLY, arranged alphabetically, with correct total affixed.

The word for dissection for this, the THIRTEENTH week of the quarter, being

"HYACINTH."

| Names. | Mar. 20. | Totals. | Names. | Mar. 20. | Totals. |
|---|---|---|---|---|---|
| Holland | 43 | 992 | Lauriston | 48 | 1004 |
| Red Cape | — | 132 | B. E. T. | — | 453 |
| Nurse Marie | — | 499 | Jacko | — | 110 |
| Garfield | — | 243 | Lady Clara | — | 258 |
| Midget | 33 | 847 | Patience | 47 | 1046 |
| E. M. S. | — | 149 | Ecila | 47 | 1031 |
| Sister | — | 591 | Maude | — | 690 |
| Duchess | — | 585 | Esperance | 49 | 1020 |
| Ellice | 53 | 1012 | Ruby | — | 698 |
| Reldas | 50 | 1049 | M. W. | 49 | 1021 |
| Jenny Wren | 51 | 911 | Crystal Palace | — | 568 |
| Mopus | — | 122 | Jackanapes | 31 | 755 |
| Neptune | — | 118 | M. M. M. | — | 25 |
| Qu'appelle | 47 | 939 | A. F. | — | 318 |
| Whitehoe | 39 | 880 | E. S. | — | 49 |
| Nurse Clague | — | 40 | | | |

### Notice to Correspondents.

N.B.—All letters referring to this page which do not arrive at **140, Strand, London, W.C.**, by the *first post on Thursdays*, and are not addressed PRIZE EDITOR, will in future be disqualified and disregarded.

